# Temporal Trends and Seasonality of Invasive Candidiasis During and After the COVID-19 Pandemic: An Interrupted Time Series Analysis in Colombia

**DOI:** 10.3390/jof12040278

**Published:** 2026-04-14

**Authors:** José M. Oñate-Gutiérrez, Carlos A. Alvarez-Moreno, Claudia Cañadas-Aragón, Hernán Vergara-Samur

**Affiliations:** 1 Clínica Colsanitas S.A., Clínica Sebastián de Belalcázar, Cali 760045, Colombia; millanonate@gmail.com (J.M.O.-G.); investigacioncsb@colsanitas.com (C.C.-A.); 2 Grupo OneHealth en Enfermedades Infecciosas, Keralty, Bogotá 111321, Colombia; 3 Clínica Colsanitas S.A., Bogotá 111321, Colombia; calvarez@colsanitas.com; 4 Fundación Universitaria Sanitas, Research Unit, Bogotá 110131, Colombia; hernan.vergara@unisanitas.edu.co

**Keywords:** invasive candidiasis, interrupted time-series, COVID-19, SARIMA, Colombia

## Abstract

Invasive candidiasis is a severe opportunistic infection whose incidence may be influenced by major disruptive events. The COVID-19 pandemic substantially altered hospital dynamics in Colombia. This study aimed to evaluate temporal trends, seasonality, and potential changes in the incidence of invasive candidiasis between 2019 and 2024. We conducted an observational time-series study using confirmed cases of invasive candidiasis from medium- and high-complexity hospitals in three major Colombian cities. Cases were aggregated quarterly. An interrupted time-series (ITS) analysis was performed. A total of 1294 cases were analyzed. An increasing trend was observed until mid-2022, followed by a decline during 2023. Seasonal decomposition revealed persistent seasonality with recurrent peaks in the second and fourth quarters. The ITS analysis did not demonstrate statistically significant changes in level or slope after the interruption (*p* > 0.05), although clinically relevant fluctuations were observed. No significant differences in temporal trends were identified across *Candida* species. Invasive candidiasis in Colombia exhibited a complex temporal evolution during and after the COVID-19 pandemic characterized by sustained seasonality and an increase followed by a decline. Although the ITS analysis did not identify statistically significant post-pandemic changes, the findings support the use of time-series models as valuable tools for epidemiological surveillance and trend monitoring.

## 1. Introduction

Invasive candidiasis represents one of the leading opportunistic fungal infections in the hospital setting, with mortality rates exceeding 40% among critically ill patients, particularly those with immunosuppression, exposure to invasive devices, or prolonged intensive care unit (ICU) stays [1,2]. Although *Candida albicans* has historically been the predominant species, recent decades have witnessed a notable epidemiological shift toward non*-albicans Candida* (NAC) species, several of which are associated with emerging antifungal resistance patterns [3,4,5].

The epidemiology of invasive candidiasis is shaped by several factors, including infection prevention and control practices, antimicrobial selective pressure, diagnostic capacity, and healthcare system strain [1,2,6]. Notably, large-scale disruptive events—such as pandemics, public health crises, or natural disasters—can substantially alter the dynamics of nosocomial infections [6], underscoring the interplay between system-level factors and infection risk.

The SARS-CoV-2 pandemic, declared in March 2020, profoundly transformed hospital organization and functionality worldwide [7,8,9]. Several studies reported a significant increase in invasive fungal infections during pandemic peaks, driven by widespread corticosteroid use, prolonged mechanical ventilation, immunosuppressive states, and healthcare system overload [10]. The incidence of candidemia in patients with COVID-19 has been reported to be up to eightfold higher than in non-SARS-CoV-2-infected patients [11]. A recent systematic review and meta-analysis including 351,268 hospitalized COVID-19 patients—55,126 of whom were admitted to ICUs—estimated a prevalence of COVID-19-associated candidemia (CAC) of 4.33% in ICUs and 2.09% among hospitalized patients overall [12]. The most frequently reported species were *C. albicans*, the *C. parapsilosis* complex, and *C. auris* [12].

Marked heterogeneity in candidemia rates across studies has been consistently observed, reflecting pre-existing variability in *Candida* epidemiology influenced by differences in incidence denominators, diagnostic capacity, and geographic distribution [12,13,14]. Notably, ICU candidemia prevalence during the pandemic was estimated to be approximately 10 times higher than in the pre-pandemic era [15]. Moreover, CAC prevalence was significantly higher in high-income countries, potentially reflecting greater antibiotic exposure, greater diagnostic availability, and differences in host susceptibility [12].

Antifungal resistance patterns observed during the pandemic have raised substantial concern. The *C. parapsilosis* complex demonstrated a sustained increase in fluconazole resistance, while *C. auris* has been reported to have an ICU prevalence of 1.22%, characterized by high nosocomial transmissibility, environmental persistence, and intrinsic multidrug resistance [16,17]. Mutations associated with emerging resistance have been increasingly identified, underscoring the importance of early detection and timely antifungal susceptibility testing to mitigate associated mortality [17]. Additionally, intensified azole use during the pandemic may have increased selective pressure on *Candida* populations, facilitating the emergence and dissemination of resistant strains [18].

Mortality associated with CAC has remained consistently high, with estimates approaching 80%, and advanced age, comorbidities, and corticosteroid exposure were identified as independent predictors of death [13,14,19]. Beyond classical risk factors, interactions between SARS-CoV-2 infection and the intestinal microbiota have been hypothesized to promote dysbiosis and microbial translocation, potentially contributing to earlier onset of candidemia in this population [14].

Despite the magnitude of this phenomenon, an important knowledge gap remains: the post-peak epidemiological evolution of invasive candidiasis has not been fully characterized, particularly in middle-income countries such as Colombia.

In this context, time series analyses provide a robust methodological framework for evaluating longitudinal epidemiological patterns and estimating the impact of disruptive events on disease incidence [20,21]. Specifically, interrupted time series (ITS) analysis enables the identification of changes in level and slope following an intervention while also accounting for underlying trends and seasonal variation [20].

The present study aimed to analyze trends, seasonality, and changes in the incidence of invasive candidiasis in Colombia between 2019 and 2024 using an ITS approach. Identifying seasonal patterns and post-pandemic fluctuations may enhance understanding of invasive candidiasis epidemiology under conditions of healthcare system stress and inform surveillance, prevention, and therapeutic strategies.

## 2. Materials and Methods

### 2.1. Study Design

We conducted a retrospective, observational, ITS study to evaluate trends in invasive candidiasis incidence in Colombia from January 2019 to October 2024, considering the COVID-19 pandemic as a potential disruptive event.

### 2.2. Study Population and Data Source

We included all confirmed cases of invasive candidiasis reported from medium- and high-complexity units in Cali, Bogotá, and Barranquilla. These institutions correspond to referral centers providing specialized care, including intensive care units, advanced diagnostic services, and management of critically ill patients.

Cases were identified and reported by hospital epidemiology teams using standardized institutional surveillance protocols based on clinical, microbiological, and coding criteria. Only cases with microbiological confirmation from sterile sites (e.g., blood cultures or other normally sterile body fluids) were included.

To minimize misclassification, colonization (defined as isolation of *Candida* spp. from non-sterile sites without clinical evidence of infection) was not considered. In cases with multiple isolates from the same patient episode, only one event per episode was included.

Due to the aggregated nature of the dataset, detailed information on individual patient characteristics, co-infections, or polymicrobial infections was not consistently available across all centers.

We aggregated data quarterly, grouping all cases per calendar quarter from the first quarter of 2019 (2019Q1) to the fourth quarter of 2024 (2024Q4). The last available observation was in October 2024, so 2024Q4 was incomplete.

### 2.3. Outcome Definition and Denominator Considerations

The primary outcome was the quarterly count of microbiologically confirmed invasive candidiasis cases. Incidence rates adjusted by hospital admissions or ICU patient-days were not available in a standardized and comparable format across participating institutions for the entire study period. Given the multicenter nature of the dataset and structural changes in hospital occupancy during the pandemic, raw case counts were analyzed to preserve internal consistency of the time series.

Because interrupted time series analysis focuses on structural changes in level and slope, aggregated case counts were considered appropriate, assuming stable case ascertainment. Seasonal decomposition and SARIMA modeling were applied to account for trends and autocorrelation.

### 2.4. Interruption Point

The interruption point was defined as the second quarter of 2020 (2020Q2), corresponding to the first COVID-19 epidemic peak in Colombia and the onset of substantial changes in national hospital dynamics, including increased ICU occupancy, changes in clinical management protocols, and intensified antimicrobial and corticosteroid use.

### 2.5. Statistical Analysis

The analysis was conducted in three complementary phases:

#### 2.5.1. Interrupted Time Series (ITS) Analysis

Segmented regression analysis was performed to evaluate changes in level and slope in the time series following the interruption point. The model was specified as follow:

Yt = β0 + β1⋅time + β2⋅intervention + β3⋅time_post + εt

where $Y_{t}$ represents the number of cases per quarter; intervention is a binary variable (0 before and 1 after 2020Q2); and time_post represents the number of quarters elapsed since the intervention.

Residual autocorrelation was assessed using the Ljung–Box test to evaluate independence assumptions of the segmented regression model.

#### 2.5.2. Additive Seasonal Decomposition

We used classical additive decomposition to break the time series into trend, seasonal, and irregular parts. This helped us spot repeated quarterly patterns and see the long-term trend more clearly.

#### 2.5.3. Seasonal ARIMA (SARIMA) Modeling

A SARIMA (1,1,1)(1,1,1)[4] model was fitted to capture the full temporal structure of the series, incorporating quarterly seasonality. Stationarity was assessed prior to model fitting, and first-order differencing was applied as required.

Logarithmic transformation of the outcome variable was considered to address potential heteroscedasticity. However, visual inspection and residual diagnostics did not demonstrate a systematic proportional increase in variance with case magnitude, and no significant heteroscedastic pattern was detected. Therefore, final models were fitted using untransformed counts to preserve clinical interpretability.

Model adequacy was assessed through residual diagnostics (Ljung–Box test and normality evaluation) and information criteria (Akaike Information Criterion [AIC] and Bayesian Information Criterion [BIC]).

### 2.6. Subanalysis

Given a clear rise in cases between the second quarter of 2022 and the fourth quarter of 2023, we conducted a focused, segmented ITS analysis during this period to assess the trend.

### 2.7. Species-Specific Analysis

Species distribution (*Candida albicans*, *C. parapsilosis*, *C. tropicalis*, and *C. glabrata* [currently classified as *Nakaseomyces glabratus*]) was analyzed separately. These species were selected as they were consistently reported in the surveillance dataset and represent the most clinically relevant causes of invasive candidiasis. Traditional *Candida* nomenclature was used for consistency with clinical and epidemiological literature. ITS models were fitted for each subgroup to assess differences in temporal trends.

### 2.8. Software and Ethical Considerations

All analyses were performed using Python 3.10 with the pandas, statsmodels, matplotlib, and pmdarima libraries. The study was approved by the Ethics Committee of Fundación Universitaria Sanitas (Protocol 143-24 UNV–Act 042-24; approval date: 29 October 2024). Only aggregated, anonymized data were analyzed, and no direct patient intervention was performed.

## 3. Results

### 3.1. General Characteristics

During the study period from January 2019 to October 2024, a total of 1294 cases of invasive candidiasis were recorded across the participating medical centers. Cases were aggregated into quarterly time points, yielding 24 observations for the time series analysis. The mean number of cases was 53.9 per quarter (standard deviation: 35.6). The regression coefficients of the interrupted time series model are presented in Table 1.

### 3.2. Seasonal Decomposition

**Figure 1:**
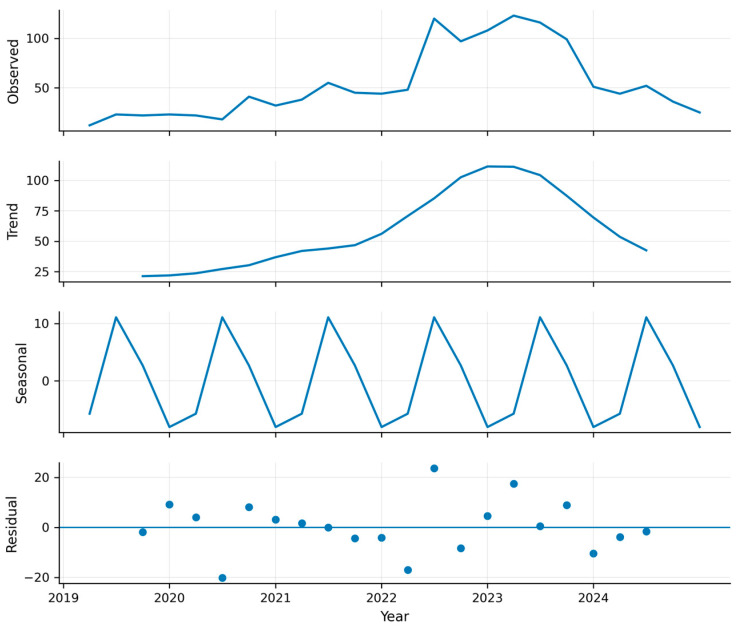
Additive decomposition of the invasive candidiasis time series in Colombia (2019–2024). The observed quarterly case counts are separated into trend, seasonal, and residual components. The seasonal component reflects a stable quarterly pattern, with recurring peaks in the second (Q2) and fourth (Q4) quarters across the study period, although these peaks may not always be visually prominent due to smoothing and variability in quarterly counts.

Additive decomposition of the time series demonstrated an increasing trend in invasive candidiasis incidence from 2019 through the second quarter of 2022 (2022Q2). This trend was followed by a sustained decline throughout 2023 (Figure 1). A stable seasonal component was identified. Recurrent peaks occurred in the second (Q2) and fourth (Q4) quarters of each year, indicating a consistent quarterly periodic pattern.

### 3.3. Interrupted Time Series Analysis

The ITS analysis did not demonstrate statistically significant changes in either the immediate level (β_2_) or slope (β_3_) following the predefined interruption point in 2020Q2 (*p* > 0.05 for both).

**Figure 2:**
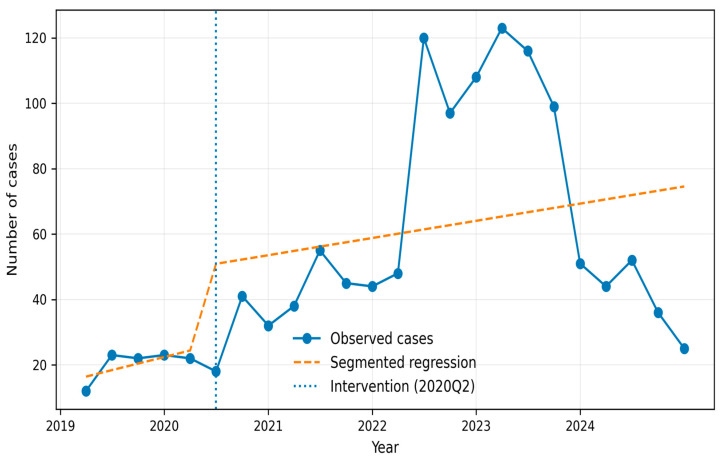
Interrupted time series analysis of invasive candidiasis cases in Colombia (2019–2024). The solid line represents observed quarterly cases, the dashed line indicates the segmented regression model, and the vertical dotted line marks the intervention point corresponding to the onset of the COVID-19 pandemic (2020Q2).

Nevertheless, the observed series showed an increase in case counts between 2021 and 2022, followed by a decline during 2023 (Figure 2).

### 3.4. SARIMA Model Results

A SARIMA model (1,1,1)(1,1,1)[4] was fitted to the observed data. The model yielded a root mean square error (RMSE) of 21.4, with AIC and BIC values of 185.5 and 190.2, respectively.

**Figure 3:**
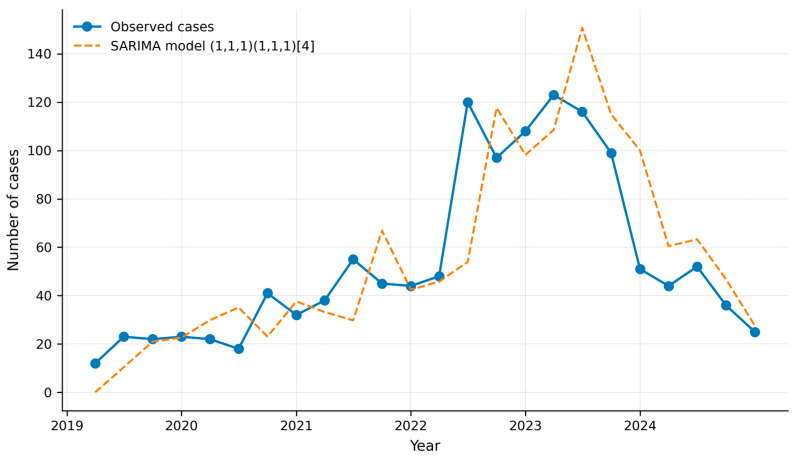
SARIMA model fit for invasive candidiasis cases in Colombia (2019–2024). The solid line represents observed quarterly case counts, and the dashed line represents fitted values from the SARIMA (1,1,1)(1,1,1)[4] model.

Residual diagnostics did not indicate significant autocorrelation (Ljung–Box test, *p* = 0.576) (Supplementary Figure S1). The fitted model captured the overall trend and quarterly seasonal pattern observed in the series (Figure 3).

### 3.5. Subanalysis (2022–2023)

During the period from 2022Q2 to 2023Q4, case counts increased followed by a subsequent decline. However, segmented regression restricted to this interval did not demonstrate a statistically significant change in slope (*p* = 0.784).

### 3.6. Species Specific Analysis Results

**Figure 4:**
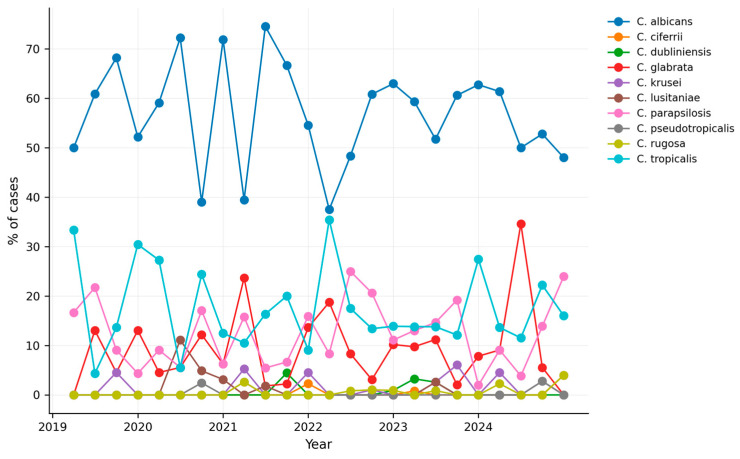
Quarterly percentage distribution of *Candida* species in Colombia (2019–2024). Each line represents the proportion of cases attributed to a given species per quarter relative to the total number of invasive candidiasis cases.

Figure 4 presents the quarterly proportional distribution of the main *Candida* species identified between 2019 and 2024. *C. albicans* remained the most frequently isolated species throughout the study period, with proportions ranging from 45% to 70% across quarters.

NAC species, including *C. tropicalis*, *C. parapsilosis*, and *C. glabrata*, were consistently detected at lower proportions, with isolated peaks observed in selected quarters. Among non-*albicans* species, *C. tropicalis* exhibited the greatest variability over time.

### 3.7. Comparative Analysis of C. albicans and Non-albicans Species

**Figure 5:**
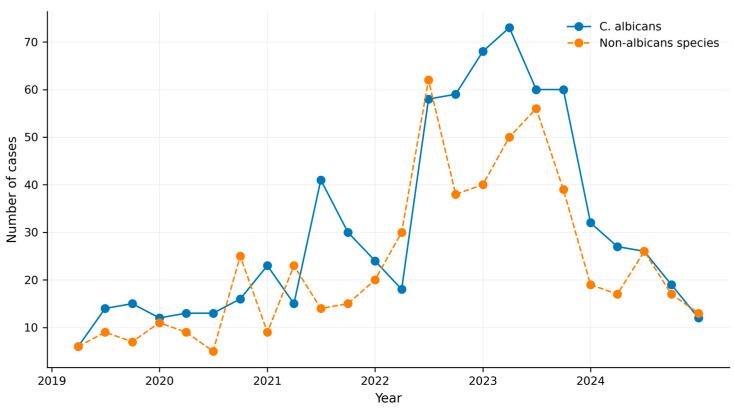
Comparative evolution of *Candida albicans* and non-*albicans* species in Colombia (2019–2024). The solid line represents quarterly cases of *C. albicans*, while the dashed line represents the aggregated number of cases caused by non-*albicans* species.

Figure 5 illustrates the quarterly absolute case counts of *C. albicans* and NAC species throughout the study period. *C. albicans* accounted for a higher number of cases in most quarters. Non-*albicans* species, although less frequent, were consistently present and demonstrated increases between 2021 and 2022. In the most recent quarters, both groups exhibited a parallel downward trend.

### 3.8. Species-Specific Interrupted Time Series Analysis

**Figure 6:**
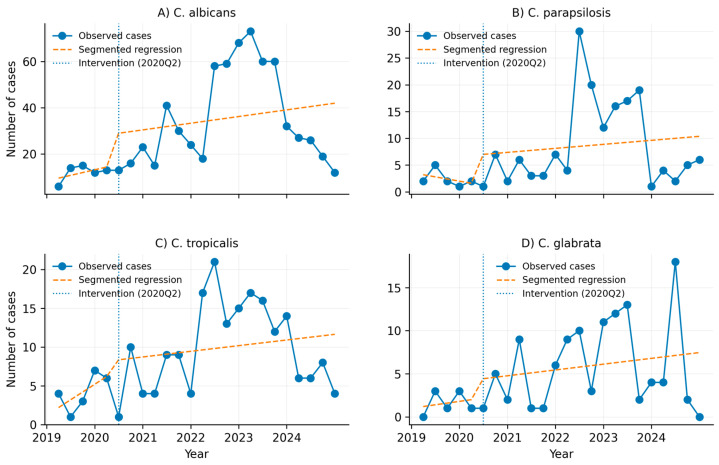
Interrupted time series analysis by *Candida* species in Colombia (2019–2024). Each panel shows the quarterly number of cases, the segmented regression model, and the intervention point corresponding to the onset of the COVID-19 pandemic (2020Q2).

Figure 6 presents the ITS analysis stratified by species for *C. albicans*, *C. parapsilosis*, *C. tropicalis*, and *C. glabrata*. Across all models, temporal fluctuations were observed without statistically significant changes in the post-interruption slope following 2020Q2 (*p* > 0.05 for all species-specific ITS models).

*C. albicans* accounted for the highest case counts throughout the study period, whereas non-*albicans* species exhibited lower absolute counts and greater interquarter variability.

### 3.9. Comparative Temporal Patterns with COVID-19

A descriptive comparison between invasive candidiasis cases and national COVID-19 incidence was performed (Supplementary Figure S2). *C. albicans* remained the predominant species during the entire study period, with higher case counts observed in 2021–2022 followed by a decline in 2023–2024. Non-*albicans Candida* species exhibited transient increases, particularly *C. parapsilosis* and *C. tropicalis* during 2022.

Although some temporal overlap with major COVID-19 waves was visually observed, no consistent parallel trend between invasive candidiasis cases and national COVID-19 case counts was identified.

## 4. Discussion

The present analysis of 1294 cases of invasive candidiasis in Colombia between 2019 and 2024 demonstrates a dynamic epidemiological pattern characterized by a sustained increase in case counts through 2022, followed by a progressive decline beginning in 2023. Although the ITS analysis did not identify statistically significant level or slope changes after the second quarter of 2020, temporal variations in trend and seasonality indicate a complex interaction between invasive candidiasis incidence and the broader healthcare context during the COVID-19 pandemic.

Our findings partially align with international reports describing abrupt increases in candidemia during the pandemic, particularly among critically ill patients, albeit with substantial heterogeneity across countries and healthcare systems [11,14,17]. The rise in case counts observed in Colombia between 2020 and 2022 coincides temporally with reports from multiple regions. In Turkey, Kayaaslan et al. documented a twofold higher incidence of ICU-acquired candidemia among COVID-19 patients compared with non-COVID patients (2.16 vs. 1.06 cases per 1000 patient-days), with earlier onset during hospitalization and significantly higher mortality (92.5% vs. 79.4%) [14].

Similarly, in Brazil, Gieburowski et al. reported an increase in candidemia incidence from 3.43 to 4.54 per 1000 admissions during the pandemic, accompanied by higher rates of sepsis, mechanical ventilation, and mortality [22]. A recent multicenter study from France identified a 1.58-fold higher risk of ICU-acquired candidemia in COVID-19 patients compared with matched non-COVID controls, even after adjustment for severity and comorbidities [23].

In contrast, our ITS analysis did not demonstrate a statistically significant structural change following the pandemic onset, despite the visually apparent increase in cases during 2020–2022. This discrepancy suggests that, in Colombia, invasive candidiasis incidence may have been influenced by multiple concurrent factors rather than a single abrupt disruption. Potential contributors include variable hospital occupancy pressures, shifts in antimicrobial stewardship policies, temporary interruptions or adaptations of antimicrobial stewardship programs (ASP), regional heterogeneity in diagnostic capacity, and fluctuations in ICU utilization.

Importantly, Colombia entered the pandemic with pre-existing antimicrobial stewardship frameworks that were further strengthened through national regulatory initiatives, alongside sustained infection prevention and control practices and microbiological diagnostic capacity. In contrast to settings where healthcare systems were overwhelmed, the absence of prolonged nationwide saturation may have enabled the sustained implementation of these measures, including rational antifungal use, species-level identification, and timely therapeutic adjustments. This could have mitigated abrupt epidemiological shifts and limited selective pressure favoring non-*albicans* species. From a public health perspective, these findings highlight the importance of resilient healthcare systems and integrated surveillance strategies that incorporate both microbiological and system-level indicators to better understand temporal dynamics. While these patterns may be relevant for settings with similar structural conditions, further studies are needed to confirm their generalizability.

The sustained decline observed in Colombia during 2023 contrasts with findings from Greece, where candidemia incidence continued to increase beyond the acute phase of the pandemic, largely driven by outbreaks of *C. auris* and fluconazole-resistant *C. parapsilosis*, with reported global fluconazole resistance rates reaching up to 41% by 2023 [24].

The downward trend in Colombia may reflect the progressive restoration of ASP and infection prevention and control measures. Previous reports have linked increases in invasive fungal infections to disruptions in infection control practices during pandemic surges, particularly for highly transmissible species such as *C. auris* and *C. parapsilosis* [24]. The re-establishment of routine infection control activities may have contributed to stabilization of incidence. Additional contextual factors could include reduced use of high-dose corticosteroids and limited exposure to immunomodulatory therapies such as tocilizumab—identified as risk factors in studies from Greece and Turkey [24,25]—as well as post-pandemic hospital reorganization, including shorter ICU stays, reduced mechanical ventilation demand, and decreased reliance on temporary critical care expansion units. Notably, no sustained nationwide outbreaks of resistant clones were identified in the aggregated Colombian data.

A consistent seasonal pattern was observed in Colombia, with recurrent peaks in the second and fourth quarters. This bimodal distribution is comparable to findings reported by Suzuki et al. within the U.S. Veterans Health Administration network, where candidemia demonstrated seasonal variation potentially associated with changes in humidity, ICU occupancy, and circulation of respiratory viral infections [26]. While causal mechanisms cannot be established in the present analysis, the persistence of seasonality suggests that underlying ecological or healthcare utilization factors may contribute to cyclical variation.

The satisfactory performance of the SARIMA (1,1,1)(1,1,1)[4] model supports the presence of regular temporal components underlying invasive candidiasis dynamics, although these may be modulated by exogenous events such as the COVID-19 pandemic. The absence of significant residual autocorrelation and acceptable predictive fit suggest potential applicability of such models for prospective surveillance in high-complexity healthcare settings.

Importantly, the stable seasonal structure further supports the notion that the COVID-19 pandemic acted as a modulator of incidence rather than a determinant of structural epidemiological change. Throughout the 2019–2024 period, *C. albicans* remained the predominant species in Colombia (45–70% per quarter), without evidence of sustained displacement toward NAC species. This pattern is consistent with findings from Belgium reported by Cugnata et al., where *C. albicans* remained the most frequent species over a seven-year period, including during the pandemic, without significant emergence of resistant *Candida* species [27].

Temporal peaks in *C. tropicalis*, *C. glabrata*, and *C. parapsilosis* during 2021–2022 reflect variability that has been reported in other geographic settings. In Greece, *C. parapsilosis* increased markedly, driven by fluconazole-resistant clones rising from 20% to 33%, alongside sustained expansion of *C. auris*, which accounted for up to 33% of all candidemia cases by 2023 [24]. Pallotta et al. reported high proportions of NAC species in ICU settings, particularly among critically ill COVID-19 patients, with mortality ranging from 39% to 46% and no significant difference between COVID and non-COVID populations [28]. Similarly, Roman-Montes and Casalini described increases in NAC species in Mexico and other countries, associated with intensified antibiotic and corticosteroid exposure during the pandemic [29,30].

In contrast to these scenarios, Colombia did not demonstrate a sustained increase in NAC species nor a structural epidemiological shift attributable to antifungal selective pressure or displacement of *C. albicans* by other species. This pattern may indicate a comparatively stable transmission ecology and potentially effective antimicrobial stewardship and infection control practices at the institutional level.

Several studies have documented the emergence and expansion of resistant *Candida* strains across diverse regions. Increased fluconazole resistance in *C. parapsilosis* has been reported in Greece and Southern Europe, together with prolonged ICU outbreaks of multidrug-resistant *C. auris* in Greece, Italy, and India [16,24,30,31]. Additionally, rising resistance among NAC species has been linked to widespread empirical azole use during the COVID-19 pandemic [17,18].

Taken together, these differences suggest that the national epidemiological profile observed in Colombia more closely resembles patterns described in Belgium than those reported in parts of the Eastern Mediterranean or South Asia. Although mortality was not evaluated in the present ecological analysis, international literature consistently reports case-fatality rates exceeding 40%, particularly among patients exposed to corticosteroids, prolonged mechanical ventilation, or septic shock [2,10,19]. The stability in species distribution and absence of documented large-scale resistant outbreaks in Colombia suggest that national mortality patterns may align with global baseline estimates (40–60%), rather than the higher mortality observed in regions affected by resistant clonal expansion. However, this inference should be interpreted cautiously in the absence of patient-level outcome data.

Overall, these findings underscore the importance of sustained local surveillance and highlight that *Candida* epidemiology during and after the pandemic appears to be strongly modulated by structural health system factors, diagnostic capacity, the burden of critical illness, and the implementation and continuity of ASP.

This study has several strengths. It includes an extended observation period spanning both pre-pandemic and post-pandemic phases, incorporates data from multiple institutions in major Colombian cities, and applies complementary analytical approaches, including ITS, seasonal decomposition, and SARIMA modeling. The combined use of these methods provides a robust framework for characterizing temporal dynamics beyond simple descriptive comparisons.

However, several limitations should be acknowledged. First, the aggregated ecological design precluded adjustment for patient-level clinical or therapeutic covariates, including severity of illness, corticosteroid exposure, mechanical ventilation, or comorbidities. Second, antifungal susceptibility testing data were not systematically available across institutions, limiting the ability to evaluate resistance trends in parallel with species distribution. Third, ITS analysis may be constrained by overlapping structural changes that are not directly observable, particularly in the context of a multifaceted healthcare disruption such as the COVID-19 pandemic. As an ecological time-series analysis based on aggregated surveillance data, this study was designed to characterize temporal epidemiological patterns rather than to assess patient-level risk factors or causal mechanisms.

Despite these limitations, the findings contribute to understanding the post-pandemic temporal dynamics of invasive candidiasis in hospital settings in Colombia and may provide insights for similar healthcare contexts. The results also underscore the importance of strengthening mycological surveillance systems with longitudinal and predictive components to support early detection of epidemiological shifts.

## 5. Conclusions

Invasive candidiasis in Colombia exhibited dynamic temporal behavior between 2019 and 2024, with an increase in cases through 2022 followed by a sustained decline in 2023. Although interrupted time series analysis did not identify statistically significant structural changes after the onset of the COVID-19 pandemic, consistent seasonal patterns and temporal fluctuations were observed.

*C. albicans* remained the predominant species throughout the study period, without evidence of sustained epidemiological displacement by non-*albicans* species. These findings highlight the importance of strengthening longitudinal mycological surveillance and incorporating predictive time series modeling into institutional early warning systems.

## Figures and Tables

**Table 1:** Interrupted time series regression model of invasive candidiasis cases in Colombia (2019–2024).

Parameter	Coefficient (β)	Standard Error	95% CI	*p*-Value
Baseline level (β_0_)	14.40	34.04	−56.61–85.41	0.677
Pre-intervention trend (β_1_)	2.00	10.26	−19.41–23.41	0.847
Immediate level change (β_2_)	25.20	29.53	−36.41–86.80	0.404
Post-intervention slope change (β_3_)	−0.69	10.35	−22.28–20.91	0.948

Abbreviations: CI, confidence interval.

## Data Availability

The data supporting the findings of this study are not publicly available due to institutional, ethical, and legal restrictions related to patient confidentiality. De-identified data may be made available from the corresponding author upon reasonable request and with permission from the Institutional Ethics Committee and Hospital authorities.

## Supplementary Materials

The following supporting information can be downloaded at: https://www.mdpi.com/article/10.3390/jof12040278/s1, Figure S1: ACF of residuals (ITS Model); Figure S2: COVID vs. Candida.

## Abbreviations

The following abbreviations are used in this manuscript:

COVID-19	Coronavirus Disease 2019
ITS	Interrupted Time Series
SARIMA	Seasonal Autoregressive Integrated Moving Average
ICU	Intensive Care Unit
NAC	Non-*albicans Candida*
SARS-CoV-2	Severe Acute Respiratory Syndrome Coronavirus 2
CAC	COVID-19–Associated Candidemia
ASP	Antimicrobial Stewardship Program
