# Supplementary figures and images for: Temporal Trends and Seasonality of Invasive Candidiasis During and After the COVID-19 Pandemic: An Interrupted Time Series Analysis in Colombia
Source: J Fungi (Basel). 2026 Apr 14;12(4):278. doi: 10.3390/jof12040278 (PMC13117731; doi:10.3390/jof12040278)

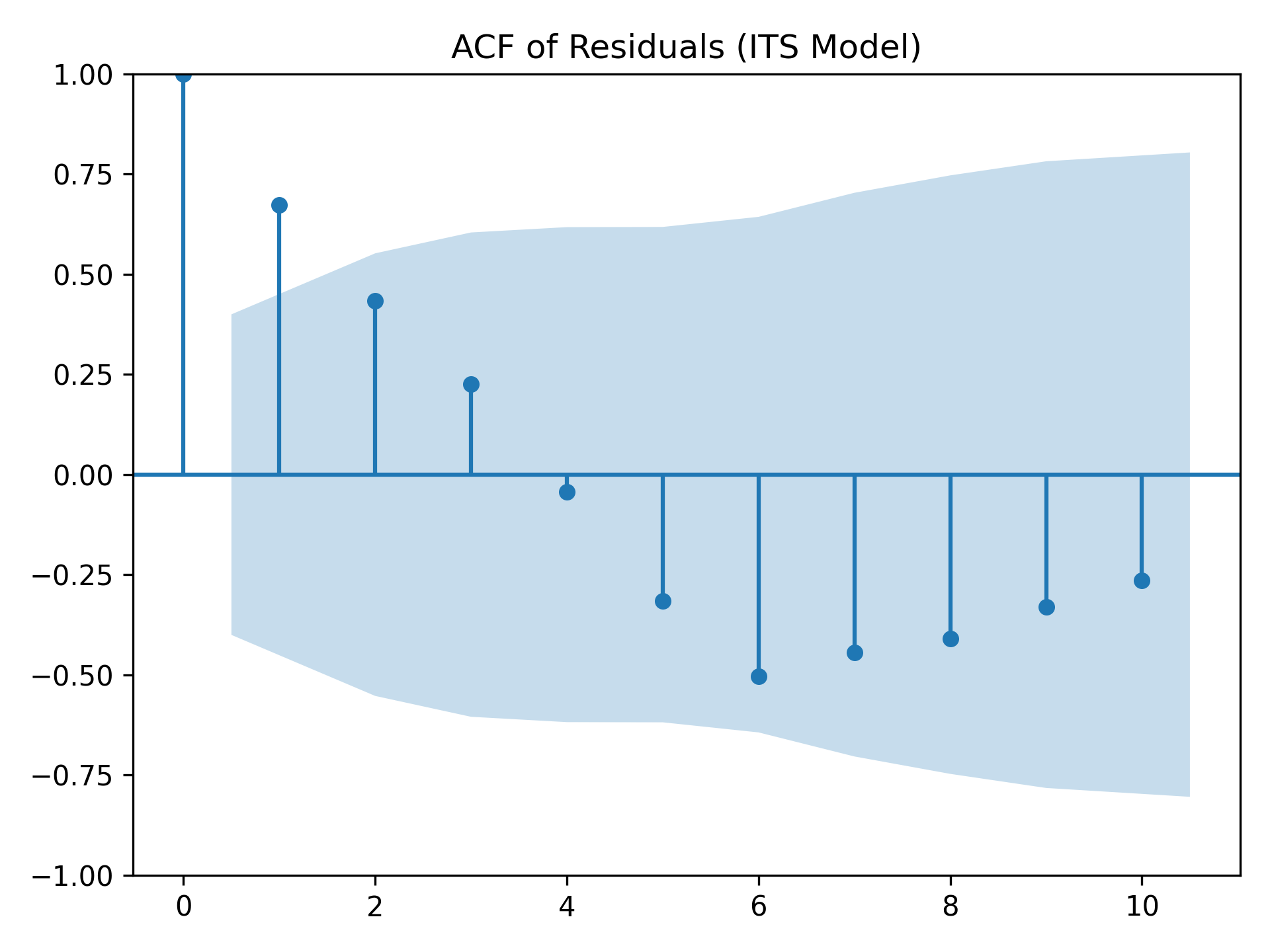

Supplement: Supplementary file 1 [file jof-12-00278-s001.zip › Figure S1. ACF of residuals (ITS Model).png]

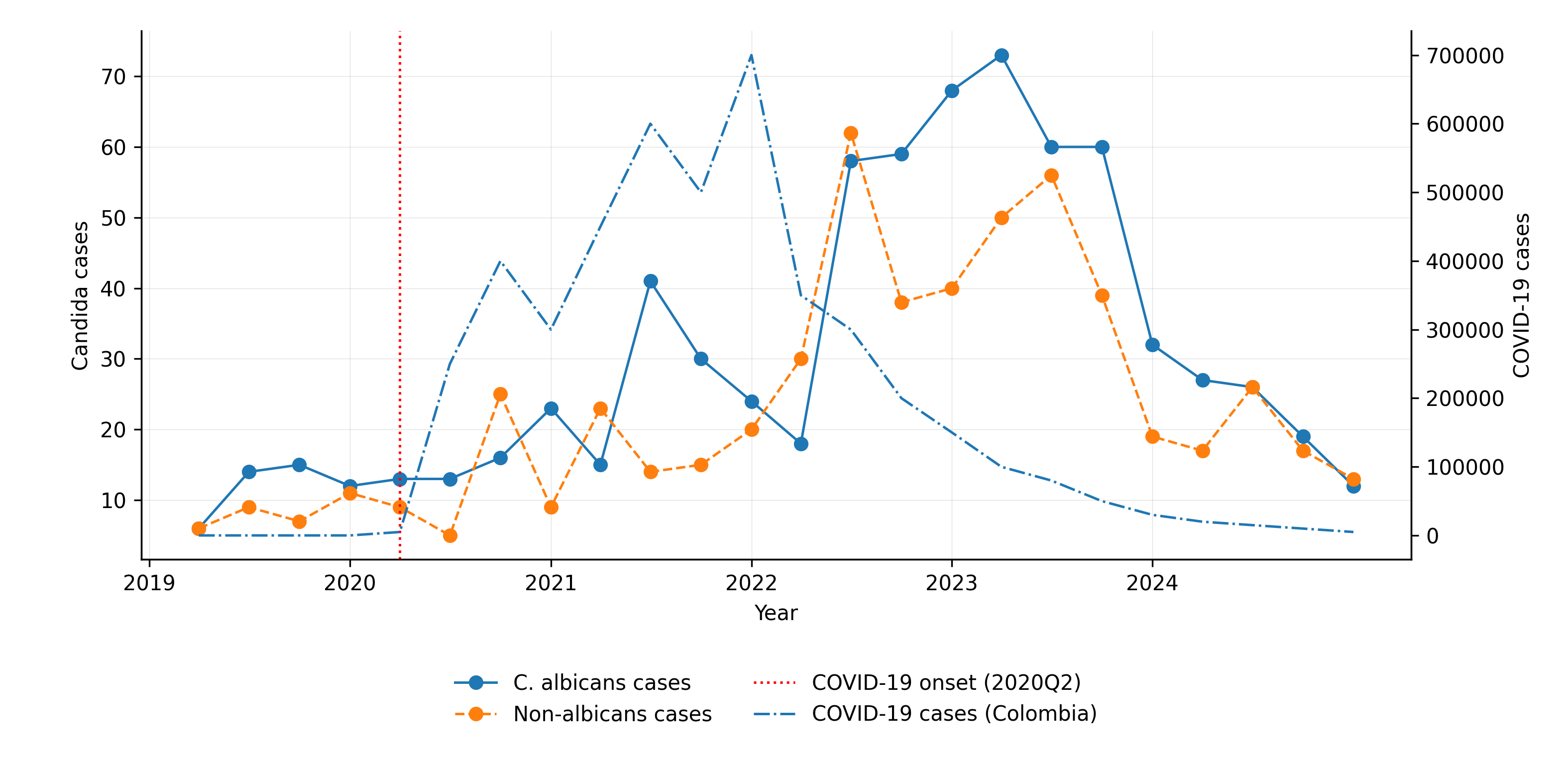

Supplement: Supplementary file 1 [file jof-12-00278-s001.zip › Figure S2. COVID vs. Candida.png]
